# Measurement of intracellular concentration of fluorescently-labeled targets in living cells

**DOI:** 10.1371/journal.pone.0194031

**Published:** 2018-04-25

**Authors:** Volodymyr Cherkas, Sergei Grebenyuk, Denys Osypenko, Alexandr V. Dovgan, Eugene O. Grushevskyi, Matthew Yedutenko, Yevhenii Sheremet, Andrew Dromaretsky, Arseniy Bozhenko, Kirill Agashkov, Nikolai I. Kononenko, Pavel Belan

**Affiliations:** 1 Department of Molecular Biophysics, Bogomoletz Institute of Physiology, Kiev, Ukraine; 2 Department of Sensory Signaling, Bogomoletz Institute of Physiology, Kiev, Ukraine; 3 Kiev Academy University, Kiev, Ukraine; Science and Technology Facilities Council, UNITED KINGDOM

## Abstract

Estimations of intracellular concentrations of fluorescently-labeled molecules within living cells are very important for guidance of biological experiments and interpretation of their results. Here we propose a simple and universal approach for such estimations. The approach is based upon common knowledge that the dye fluorescence is directly proportional to its quantum yield and the number of its molecules and that a coefficient of proportionality is determined by spectral properties of the dye and optical equipment used to record fluorescent signals. If two fluorescent dyes are present in the same volume, then a ratio of their concentrations is equal to a ratio of their fluorescence multiplied by some dye- and equipment-dependent coefficient. Thus, if the coefficient and concentration of one dye is known then the concentration of another dye can be determined. Here we have demonstrated how to calculate this coefficient (called a *ratio factor*) and how to use it for concentration measurements of fluorescently tagged molecules. As an example of how this approach can be used, we estimated a concentration of exogenously expressed neuronal Ca^2+^ sensor protein, hippocalcin, tagged by a fluorescent protein in a dendritic tree of rat hippocampal neurons loaded via a patch pipette with Alexa Fluor dye of known concentration. The new approach should allow performing a fast, inexpensive and reliable quantitative analysis of fluorescently-labeled targets in different parts of living cells.

## Introduction

Fluorescence microscopy is commonly used for the qualitative analysis of protein distribution in fixed and living cells and organisms. Over the last two decades, real time imaging techniques used to study a role of various molecules in living cells have become well established and commonly used in a laboratory practice. The groundbreaking event in the protein distribution studies was the discovery and further refinement and application of green fluorescent protein (GFP). Genetically encoded fluorescent reporter-tagged proteins have been developed as a tracing tool for localization of expressed proteins within a cell [1,2]. The proteins tagged with a fluorescent reporter can be traced to determine their localization as well as their mobility within the cell in real time [1,3]. However, estimations of localization and mobility of proteins are not the only demands in the field and many tasks require genuine quantification of intracellular protein concentrations. One of the main issues in studies using genetically-encoded proteins, is the estimation of the expression level of the exogenous protein construct, compared to that of the corresponding endogenous one. Addressing this issue is a prerequisite for proper interpretation of experimental results since overexpression of exogenous proteins may shift interaction between the protein under study and its counterparts leading to modifications of involved signaling pathways. Several approaches have been proposed to estimate the concentration of fluorescent proteins at a single cell level [4,5]. These approaches have been mainly based on usage of recombinant bacterially expressed proteins not always available for a particular study. To estimate the exogenous fluorescent protein concentration expressed in a cell using these approaches, its fluorescence intensity is compared with one of recombinant protein contained in artificial objects geometrically similar to cell soma. Estimations obtained with these approaches rely upon suggestion that fluorescent properties and distribution of recombinant (localized in an artificial object) and exogenous (localized in a cell soma) proteins are similar. In most cases these suggestions are not well grounded due to differences in pH and metal ions present, post-translational modifications, as well as homogeneous vs heterogeneous distribution of the fluorescent proteins in the test volume and the cell. Therefore, substantial errors can be expected in the estimations.

Besides, these approaches require additional efforts to produce objects enriched with recombinant fluorescent proteins and do not allow for measurements in subcellular compartments. Thus, despite the fast-paced progress of imaging techniques, the means for quantitative analysis of protein concentration at a single cell and subcellular level remain underdeveloped.

In the present work, we propose a simple and effective method for the measurement of intracellular concentration of fluorescently labeled molecules. The method exploits a simple fact that the fluorescence detected from a preparation depends on a) concentration and optical properties of fluorescently tagged molecules and b) optical function (i.e., spectral properties) of equipment, which is used to measure the fluorescent signal from the preparation. Knowing the optical functions of light source, filters, detectors, objective, etc., and loading a cell with a reference dye of known concentration and quantum yield, it is possible to calculate the concentration of the fluorescently tagged molecules including exogenously expressed fluorescent proteins. The new method is especially suitable in electrophysiological research, since patch clamp recordings is an ideal tool to load a cell with the reference dye of known concentration.

## Methods

### PC12 cells

Undifferentiated PC12 cells were obtained from the Cell Culture Bank of National Academy of Sciences of Ukraine (Bogomoletz Institute of Physiology, Kiev, Ukraine). The PC12 cells were cultured on round glass coverslips in DMEM supplemented with 10% fetal bovine serum, 5% horse serum and 0,25% gentamycin in the absence of a nerve growth factor. The cells were maintained in 12-well culture dishes at 37˚C, in a 5% CO_2_ humidified atmosphere. The culture medium was changed every 3–5 days and cells were split when necessary. Cells were transfected at ∼75% confluence with 0.3–0.5 μg of DNA per well using Lipofectamine 2000 (Thermo Scientific, USA) and then cultured for 1–3 days until they were used in experiments.

### Hippocampal culture

All procedures used in this study were approved by the Animal Care Committee of Bogomoletz Institute of Physiology (Kiev, Ukraine) and conform to the Guidelines of the National Institutes of Health on the Care and Use of Animals. Hippocampi were obtained from newborn Wistar rats (age postnatal day 0–1) killed via rapid decapitation without anaesthesia. All rats were from the vivarium of Bogomoletz Institute of Physiology (Kiev, Ukraine). Hippocampi from newborn rats were enzymatically dissociated with trypsin. Cell suspension at the initial density of 3–5 *10^5^ cells per cm^2^ was plated on glass coverslips coated with laminin and poly-D-lysine. Cells were maintained in 12-well culture dishes in a feeding solution consisted of minimal essential medium, 1% horse serum and other necessary additives in humidified atmosphere containing 5% CO_2_ at 37°C as described previously [6]. Neurons were transfected with 0.9–1.1 μg of DNA per well using Lipofectamine 2000 (Thermo Scientific, USA) and then cultured for 2–3 days until they were used in experiments.

### Plasmids

Cerulean-Venus tandem (CTV) construct, Enhanced Cyan Fluorescent Protein (ECFP), and Hippocalcin tagged by Enhanced Yellow Fluorescent Protein (HPCA-EYFP) were amplified from a construct described previously [7,8].

### Fluorophore parameters

A quantum yield is a fluorophore parameter, which is determined in conditions when fluorophore molecules do not interact with each other or with other fluorophores. If such conditions are preserved, quantum yields of particular fluorophore obtained from the literature can be used for calculations of fluorophore concentrations according to Eq 2. In the CTV construct, the Cerulean domain is separated from the Venus domain by a 229 amino acid linker encoding the TRAF domain of human TRAF2 [9]. The crystal structure of a TRAF domain has been solved and predicts at least an 8 nm distance between Cerulean and Venus in the CTV construct [9]. The interaction between Cerulean and Venus at such a distance should be minimal [10,11] resulting in a low FRET within each pair of Cerulean and Venus. However, the structure of TRAF2, used in CTV, shows that it exists as a mushroom-shaped homotrimer [9,12] and an excited Cerulean transfers energy to not one but three potential acceptors leading to a measurable FRET efficiency of 8±5% [12]. This additional energy transfer to Venus results in an effective increase of Venus quantum yield from 0.57 to 0.62 (0.57*1.08). Three Cerulean donors within one homotrimer are predicted to be in close proximity that results in substantial homo-FRET between three Cerulean fluorescent proteins and an energy migration from them of 19±5% [12] producing a decrease in Cerulean quantal yield by the same value. Thus, quantum yields for Cerulean is decreased in CTV from 0.57 [13] to 0.50 (0.57*(1–0.19)). Thus, quantum yields of 0.50 for Cerulean and 0.62 for Venus were used for the following calculations.

The relative brightness of Venus (a product of an extinction coefficient by a quantum yield) is pH-dependent and it has been previously obtained at pH 7.0 [14]. In this work, the value for Venus relative brightness was corrected taking into account that pK_a_ (logarithm of dissociation constant for H+, K_d_) for Venus is 6.0 and that pH of intracellular solution used in our experiments was 7.3. Thus, a deprotonated part of Venus was larger at pH 7.3 compared to pH 7.0. This increase in the deprotonated part of Venus can be calculated based on law of mass action written in the following way:

$$[V]/[V_{0}]=K_{d}/(K_{d}+[H]),$$

where [*V*] is a concentration of deprotonated Venus, [*V*_0_] is a total Venus concentration, [*H*] is a hydrogen concentration. Calculations based on this formula demonstrate 5% increase in the deprotonated part of Venus at pH 7.3 leading to about 5% higher Venus quantum yield. Thus, the final value of Venus quantum yield was set to 0.65 (0.62*1.05).

Extinction coefficients and quantum yields for other used fluorescent proteins and dyes were obtained from Nikon [13] and Thermo Fisher Scientific [15] web sites.

### Electrophysiological recordings

PC12 cells and neurons growing in cultures were visualized using inverted microscopes (IX70 or IX71; Olympus, Tokyo, Japan). Whole-cell patch-clamp recordings in either current- or voltage-clamp mode were performed using an EPC-10/2 amplifier controlled by PatchMaster software (HEKA, Freiburg, Germany).

The composition of the extracellular solution was as follows (mM): NaCl 150, KCl 2, CaCl_2_ 2, MgCl_2_ 1, HEPES 10, glucose 10, pH 7.3, osmolarity 320 mOsm. Experiments involving the hippocampal neurons were carried out in the presence of d-2-amino-5-phosphonopentanoic acid (APV, 40 μm). The intracellular solution contained (mM): Methansylfonic acid 135, KCl 10, MgATP 4, EGTA 1, Na_2_GTP 0.4, HEPES 10, Phosphocreatine 5, pH 7.3 with KOH, osmolarity 290 mOsm. In some experiments, the intracellular solution was supplemented with Oregon Green 488 and Alexa Fluor 594 dye. Patch electrodes were pulled to obtain a resistance of 3–5 MΩ. Membrane voltage or transmembrane current recordings were low-pass filtered (3 kHz) and acquired at 10 kHz. Recordings with a leak current > 200 pA and access resistance > 25 MOm were discarded. An access resistance was measured during a time course of experiments in order to control cell dialysis and to ensure complete wash-in of reference label via an intracellular perfusion.

All experiments were conducted at room temperature.

### Fluorescence measurements

Time-lapse imaging of PC12 cells and hippocampal neurons transiently transfected with fluorescent protein(s) and/or loaded with fluorescent dyes was performed using a TILL Photonics wide-field imaging system (TILL Photonics, Gräfelfing, Germany) controlled by TILLvision software and installed on inverted microscopes (IX70 or IX71, Olympus, Japan) equipped with oil-immersion objectives (40 ×, NA 1.35 or 60 × NA 1.25; Olympus, Japan). Monochromator based excitation allowed to measure fluorescence of labels used in most experiments without a substantial cross-talk between them. In case of a substantial cross-talk between fluorophores (e.g. Alexa Fluor 594 fluorescence in EYFP channel (enhanced yellow fluorescent protein), images in an emission EYFP channel used for calculation were taken before neurons under study are patched and filled with Alexa Fluor 594. Since EYFP is not excited at 594 nm, images in the emission Alexa Fluor 594 channel after loading of the neurons contain no contribution of EYFP fluorescence, thus far resulting in an efficient discrimination of Alexa Fluor 594 and EYFP fluorescence.

In experiments with a Cerulean-Venus tandem, 5 pairs of images were acquired for each cell and images for each fluorescent protein (FP) were averaged. These averaged images were used for validation of the suggested approach.

In case of patch clamp recordings, time-lapse imaging was started 1–3 min before membrane rupture. Fluorescent protein and dye fluorescence were recorded at a slow acquisition rate in a range of 0.03–0.1 Hz in order to minimize photobleaching. Regions of interest (ROIs) were chosen in soma and dendrites of cells under study and averaged values of fluorescence in the ROIs were calculated in TILLvision software for all fluorescent labels used in particular experiments and plotted as a function of time.

### Estimating the mobile and immobile fractions of cytoplasmic FPs

Initially, the image of the cell is acquired (in the target fluorophore imaging channel) before starting the patch clamp recordings. Then, the cell is patched and allowed to perfuse for several minutes, during which time free intracellular fluorophore is washed out and the only fluorescent signal observed afterwards corresponded to the immobile fluorophore fraction. In order to estimate the cytosolic concentration of a target fluorophore, the image acquired after the target fluorophore washout is subtracted from the image acquired right before a membrane rupture. The resulting image is representing the mobile (i.e. cytosolic) fraction of the target fluorophore and can be used for quantification of the cytosolic target FP concentration based on Eq 2. This approach also allows to quantitatively estimate immobile fraction of FP under study.

### Statistics

Quantitative results are presented as mean or median values as indicated in the text.

### Chemicals

All chemicals used for cell culturing were purchased from Thermo Scientific (Ukraine). All other chemicals were purchased from Sigma (Germany) except Alexa Fluor 594 purchased from Life Technologies (USA).

## Results

### Measurements of concentration of fluorescently labelled molecules

The fluorescence intensity detected from a fluorescent label is brought to a correspondence with its concentration by the expression [16]:

$$F=E_{ex}E_{em}V[L],$$

where *E*_*ex*_, *E*_*em*_ are excitation and emission functions of the corresponding light paths, *V* is a sample volume and [*L*] is the label concentration.

In order to calculate *E*_*ex*_ and *E*_*em*_, spectral properties of each optical element in the corresponding light path of the imaging systems (Olympus IX70/71 microscopes) along with absorption/emission properties of the fluorescent label have to be taken into account. Then [17],

$$E_{ex}=I\varepsilon \int_{\lambda_{1}}^{\lambda_{2}}S_{src}(\lambda )S_{slit}(\lambda )S_{ex}(\lambda )({1-S}_{dichr}(\lambda ))S_{obj}(\lambda )S_{abs}^{L}(\lambda )d\lambda$$

where [*λ*_1_,*λ*_2_] is a full *excitation* spectral band of monochromator of imaging system, *S*_*src*_(*λ*) is the spectrum for the light source, *S*_*ex*_(*λ*) is the excitation filter spectrum, *S*_*dichr*_(*λ*) is the dichroic mirror spectrum, *S*_*obj*_(*λ*) is the objective transmission spectra, and $S_{abs}^{L}(\lambda )$ is the normalized fluorescent label absorption spectrum; *ε* is the extinction coefficient of the fluorescent label. *I* is the intensity of the light source at the maximum of its spectrum. As it will be shown below, *I* is reduced in the calculations and therefore does not have to be determined.

The emission function of the emission light path [17]:

$$E_{em}=Q\int_{\lambda_{3}}^{\lambda_{4}}S_{em}^{L}(\lambda )S_{obj}(\lambda )S_{dichr}(\lambda )S_{em}(\lambda )S_{det}d\lambda$$

where [*λ*_3_,*λ*_4_] is the full spectral band for *emission* detection, *Q* is the quantum yield of the fluorescent label, $S_{em}^{L}(\lambda ),S_{obj}(\lambda ),S_{dichr}(\lambda ),S_{em}(\lambda ){,S}_{det}$ are spectra of the fluorescent label emission, objective transmittance, dichroic filter transmittance, emission filter transmittance and normalized detector sensitivity, correspondingly. $S_{em}^{L}(\lambda )$ should be normalized to yield an area under $S_{em}^{L}(\lambda )$ equal to 1. In this case a product of quantum yield, *Q*, and *S*_*em*_(*λ*) integrated in a range of emission wavelengths would give the quantum yield: $\int_{\lambda_{3}}^{\lambda_{4}}QS_{em}^{L}(\lambda )d\lambda =Q$.

Dividing the fluorescence intensity of the target label *F*^*tar*^ (to be determined) by fluorescence intensity of the reference label *F*^*ref*^ (of known concentration) located in the same volume *V*, we obtain:

$$\frac{F^{tar}}{F^{ref}}=A\frac{\left[L^{tar}\right]}{\left[L^{ref}\right]},A=\frac{E_{ex}^{tar}E_{em}^{tar}}{E_{ex}^{ref}E_{em}^{ref}},$$ (1)

where [*L*^*tar*^] and [*L*^*ref*^] are concentrations of target and reference labels, respectively; *A* denotes a coefficient, which is dependent on the equipment and label optical properties, which we will call *a ratio factor*. The value *V* is reduced and is not necessary to be determined.

Thus, the target protein concentration can be estimated as:

$${[L}^{tar}]=\frac{1}{A}\frac{F^{tar}}{F^{ref}}[L^{ref}]$$ (2)

Usually, spectral properties of the optical equipment necessary to calculate the ratio factor are deemed unknown; however, from our experience, spectral data are shipped along with the corresponding equipment, except, probably, for the objectives. The data for objectives were obtained from the manufacturer (Olympus, Germany). In general, approximate equipment spectral properties can be found on manufacturer’s web-sites or directly obtained from manufacturers of the optical equipment. Thus, the *ratio factor A* can be calculated for the particular optical imaging system and particular pair of fluorescent labels using relatively simple calculations (Eq 1) and can be further used for the estimation of target label concentration if the concentration of reference label is known (Eq 2).

In some cases, when a concentration ratio of the labels is *a priori* known the *ratio factor A* can be immediately obtained from the Eq 2. This is possible, for example, if a tandem of fluorescent labels is expressed in a cell or equal concentrations of fluorescent dyes are loaded into a cell (e.g. a combination of a morphological tracer and Ca^2+^ dye). Then the label concentration ratio is 1 and the ratio factor can be calculated as a simple ratio of target and reference fluorescence:

$$A=\frac{F^{tar}}{F^{ref}}$$ (3)

directly from the experimental data.

### Validation of the method and estimation of associated error

#### Validation of the method using fluorescent tandem construct

In order to experimentally validate that the *ratio factor A* can be correctly obtained from the Eq 1 using spectral properties of optical equipment and fluorescent labels and in order to estimate accuracy of the method we employed a fluorescent tandem construct consisting of two fluorescent labels, namely two fluorescent proteins, Cerulean and Venus, connected by a long aa linker to avoid FRET between the labels [7]. By using the tandem, the expected concentration ratio of the fluorescent labels was set to 1. Therefore, if our experimental arrangement and general reasoning are adequate and the manufacturer supplied spectral data are precise enough, then (according to Eqs 2 and 3) an apparent concentration ratio, *ξ*, obtained from the Eq 1,

$$\xi =\frac{\left[L_{V}^{tand}\right]}{\left[L_{C}^{tand}\right]}=\frac{1}{A}\frac{F_{V}^{tand}}{F_{C}^{tand}}$$ (4)

should be equal to 1. Here, $F_{C}^{tand}$ and $F_{V}^{tand}$ are the fluorescence recorded from Cerulean and Venus of the tandem construct.

**Fig 1:**
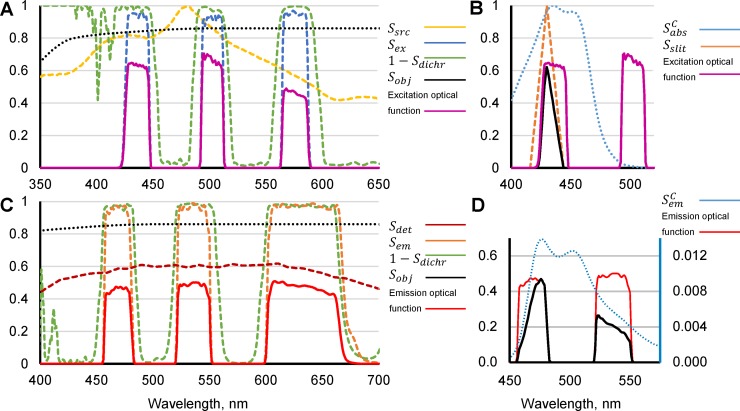
Parameters necessary for calculation of *ratio factor A* for Cerulean and Venus fluorescent proteins. (A) Spectra of the optical components in the excitation path of the imaging system. *S*_*src*_(*λ*), *S*_*ex*_(*λ*), *S*_*dichr*_(*λ*), *S*_*obj*_(*λ*) are spectra for the light source (Polychrome V monochromator, TILL Photonics, yellow), excitation filter (Chroma 69008x, blue), dichroic mirror (Chroma 69008bs, green) and objective (Olympus UAPO 40XOI3/340, black), respectively, mounted on Olympus IX71 microscope. Point-by-point multiplication of spectra for each optical element yields an optical function of microscope excitation light path shown by a violet trace. This excitation optical function of the particular imaging system can be used for calculation of a *ratio factor A* for a wide range of different fluorescent labels. (B) Further point-by-point multiplication of the optical function (violet), normalized Cerulean absorption spectrum $\left(S_{abs}^{C}(\lambda \right)$ (blue), and a spectrum of monochromator slit chosen for a given experiment (orange) gives a function of excitation path for Cerulean (black). Integration of this function and multiplication by the extinction coefficient for Cerulean absorbance results in $E_{ex}^{C}$, necessary for estimation of the *ratio factor A* (see Eq 1). (C) Spectra of the optical components in the emission path of the imaging system. *S*_*obj*_(*λ*),*S*_*dichr*_(*λ*),*S*_*em*_(*λ*),*S*_*det*_(*λ*) are objective (Olympus UAPO 40XOI3/340, black), dichroic mirror (Chroma 69008bs, green), and emission filter (Chroma 69008m, dotted red) transmittance and detector (QImaging ExiBlue, brown) sensitivity, respectively. Point-by-point multiplication of spectra for each optical element gives an optical function of microscope emission path shown by a red bold trace. This emission optical function of the particular imaging system can be used for calculation of a *ratio factor A* for a wide range of different fluorescent labels. (D) Further point-by-point multiplication of the optical function (red dashed trace; left Y axes) and Cerulean emission spectrum, integral of which is normalized to 1, $\left(S_{em}^{C}(\lambda )\right)$ (blue dashed trace; right Y axes) gives the function of emission path for Cerulean (black trace, right Y axes). Integration of this function and multiplication by Cerulean quantum yield results in $E_{em}^{C}$.

In order to compare the expected and apparent concentration ratio for Cerulean and Venus, fluorescence of both labels, $F_{C}^{tand}$ and $F_{V}^{tand}$, was recorded from soma of PC12 cells transiently transfected to express the tandem. Calculations of *ratio factor A* were performed based on spectral data of the optical elements used in our imaging setup (Fig 1A and 1C) and quantum yields, extinction coefficients (see Fluorophore parameters in Methods) and spectra [18] of Cerulean and Venus (Fig 1B and 1D). All items constituting the light path of used imaging system are listed in Table 1 together with a description of how the spectral data can be obtained if not available in a laboratory. Fluorescence spectra for Cerulean and Venus as well as their extinction coefficients and quantum yields are publicly available from many internet sites [13,18,19]; necessary data about these and other fluorescent proteins and fluorescent labels can be also obtained from their providers.

**Table 1:** Reference data on optical elements of the imaging system.

Item	Model	Reference for spectrum
1. Light source	FEI Polychrome V	on request from FEI-Munich
2. Excitation transmittance filter	Chroma 69008x	www.chroma.com
3. Dichroic mirror	Chroma 69008bs	www.chroma.com
4. Detector	PCO Sensicam VGA	www.pco.de
5. Emission transmittance filter	Chroma 69008m	www.chroma.com
6. Objective	Olympus UAPO 40XOI3/340	on request from Olympus

Calculations for $E_{ex}^{V}$ and $E_{em}^{V}$ for Venus were performed as shown above for Cerulean. The *ratio factor A* was calculated based on Eq 1 using the obtained values for $E_{ex}^{V}$, $E_{em}^{V}$, $E_{ex}^{C}$, and $E_{em}^{C}$.

Thus, according to Eq 1, the *ratio factor A* can be calculated as:

$$A=\frac{E_{ex}^{C}E_{em}^{C}}{E_{ex}^{V}E_{em}^{V}}.$$

The calculations of *E* terms for the corresponding light path and fluorescent label were performed by point-by-point multiplication of the respective optical spectra (Fig 1) and integration over black traces in Fig 1B and 1D.

The *λ* range for each *E* was chosen to encompass all spectral values of the constituting spectra. Where not defined for some *λ*, spectral values were padded with zeroes, so that all spectra have the same number of data points. After calculation, the *ratio factor A*, for a particular Olympus IX71 microscope and Cerulean and Venus fluorescent labels was found to be 2.77.

**Fig 2:**
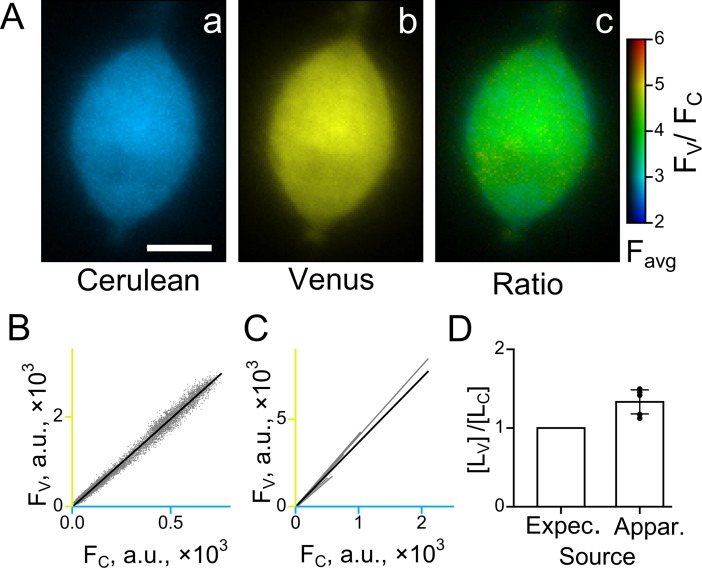
Estimation of approach accuracy using a tandem of Cerulean and Venus. (A) Images of representative PC12 cell expressing the tandem: Cerulean fluorescence (a), Venus fluorescence (b), and a ratio image of Venus to Cerulean fluorescence (c). A color and intensity of each pixel in (c) represent the ratio of Venus/Cerulean fluorescence and averaged intensity of respective pixels in the images (a) and (b) *(F*_*avr*_ *= (F*_*C*_ *+ F*_*V*_*)/2)*, respectively. A scale bar in (a) is 5μm. (B) A linear regression of correlation plot between Cerulean and Venus fluorescence intensities for each pixel within the PC12 cell image shown in (A). A strong linear correlation between the intensities (slope = 3.922±0.004, intercept = 11.1±0.01, R^2^ = 0.99; the slope is significantly different from zero at the 0.05 level) demonstrates co-localization of fluorescent protein labels. (**C**) Linear regressions of correlation plots similar to one shown in (B) for five PC12 cells. Cells having different levels of tandem expression were chosen for this plot in order to demonstrate that the ratio of fluorescence intensities remains unchanged in the wide range of tandem expression levels. (D) Expected (Expec.) and apparent (Appar.) ratios of Venus to Cerulean concentrations in the tandem ([*L*_*V*_]/[*L*_*C*_]). The histogram demonstrates that the apparent ratio of Venus to Cerulean concentrations estimated based on the *ratio factor* (1.33±0.06, mean±S.E.M., n = 5) is close to the expected ratio, which is equal to 1. It indicates that an error associated with inaccurate determination of spectral properties of labels and equipment is about 30%.

In order to estimate an apparent concentration ratio *ξ* based on the *ratio factor*, a ratio of fluorescent intensities has also to be calculated (Eq 4). Cerulean and Venus are a pair of fluorescent proteins that is well suited for the current study since their cross-talk is minimal [13]. If the fluorescent labels used have a substantial cross-talk, their genuine fluorescence intensities necessary for the calculation of fluorescence ratio can be obtained by linear unmixing [10,11,20]. Thus, a ratio of fluorescent intensities can be directly calculated from images of PC12 cells expressing the tandem obtained at excitation wavelengths close to maxima of Cerulean and Venus (433 nm and 515 nm, respectively). Fig 2 demonstrates that the fluorescent intensity ratios obtained from linear regression of Cerulean to Venus fluorescence are almost the same for different PC12 cells (3.69±0.22 (mean±S.E.M.), n = 5). Correlation between the fluorescent intensities is strong (R^2^>0.94 for all tested cells; n = 5) and is independent upon protein expression level, location inside the cells and particular culture used in experiments (Fig 2B and 2C). This confirms that the fluorescence ratio faithfully represents the ratio of concentrations for Cerulean and Venus. It is also important to note that the strong spatial linear correlation reflects strong co-localization of the labels.

The apparent concentration ratio *ξ* was then calculated based on the obtained *ratio factor* and label fluorescence ratio:

$$\xi =\frac{\left[L_{V}^{tand}\right]}{\left[L_{C}^{tand}\right]}=\frac{1}{A}\frac{F_{V}^{tand}}{F_{C}^{tand}}=\frac{1}{2.77}3.69=1.33$$

The obtained apparent concentration ratio deviates from 1, indicating to some inaccuracy of the method. This inaccuracy most probably occurs due to differences between Cerulean and Venus fluorescent parameters and spectra in the intracellular environment and calibrating solutions. In particular, quenching or environmental- and concentration-dependent changes in quantum yield, folding and maturation efficiency of fluorescent proteins, spectral shifts, and FRET can change both parameters and spectra. Despite that, the demonstrated ~30% accuracy of the approach for two fluorescent proteins employed can be considered practically useful for most biological applications. First, it allows, for the first time, to measure and compare protein expression levels in the neighboring cells within the same preparation as well as dynamics of protein expression or translocation at cellular and subcellular levels. Second, in spite of 30% error in estimations of protein concentrations, the obtained estimations will still allow to predict protein signaling processes since the behavior of a cellular pathway is not expected to be greatly altered by 30% changes in the protein expression level. Moreover, the accuracy of estimations can be substantially improved by a usage of (i) organic dyes (see the next part of the Results) and/or (ii) exact spectra of fluorescent proteins, measured within cells, instead of relying on spectra obtained from suppliers’. In the latter case, any environment-dependent modifications of fluorescent label spectra can be corrected.

On the other hand, the apparent concentration ratio *ξ* obtained for two particular fluorescent labels may be used as a correction coefficient to the *ratio factor* in a given experimental arrangement that includes the chosen fluorescent labels:

$$\left[L^{tar}]=\frac{1}{\xi A}\frac{F^{tar}}{F^{ref}}[L^{ref}\right]$$ (5)

giving, in this case, practically accurate estimation of target label concentration.

#### Validation of the method using two organic dyes

The tandem measurements described above are grounded on certain theoretical assumptions, such as estimation of homo-FRET, direct FRET, possible number of subunits in CTV homomer, etc., which are not completely proven in given experimental conditions. In order to provide the validation of the method and its associated error, we performed a series of similar experiments, this time with two organic dyes of equal concentrations loaded into a cell via glass micropipette. Oregon Green 488 and Alexa Fluor 594, used in these experiments, have a low crosstalk if excited at 498 nm and 582 nm respectively: Oregon Green 488 was not excited at 582 nm, and Alexa Fluor 594 had only 7% excitation (compared to its maximum) at 498 nm. Hippocampal cultured neurons were used in this set of experiments in order to additionally demonstrate that the suggested approach is applicable to different cell types.

In order to load the dyes into the cells we employed standard patch clamp technique in a whole-cell configuration. It is commonly accepted that the cell somatic cytosol is completely exchanged with an intra-pipette solution within several minutes after the whole-cell configuration is established. It is the essence of this methodology, which is also called an intracellular perfusion technique, that the soluble composition of intracellular milieu and pipette is the same [21,22]. Therefore, the reference dye and other dissolved molecules are expected to reach an even distribution within the cell and the pipette. Since the volume of the pipette is many orders of magnitude larger than the cell volume, it is assumed that the equilibrated concentration of the dyes in a cytosol will be identical to their original concentration in the pipette.

Cells (n = 8) were initially patched with pipettes containing 100 μM of each dye in a cell-attached mode (without rupturing the plasma membrane) and wide field fluorescence recordings in Oregon Green 488 and Alexa Fluor 594 emission channels were immediately started at the rate of one frame per 15 s. After taking 3 frames the membrane of patched cell was ruptured allowing the dyes to perfuse into the cell cytosol. In 5–10 min after the rupture both Oregon Green 488 and Alexa Fluor 594 fluorescence intensities reached steady-state levels indicating that the dye concentrations in the pipette and cytosol became equilibrated. After that, a focal plane was moved 50 μm upward in order to take fluorescent images of both dyes in the pipette, far from the cell soma.

First, we tested whether the perfusion of dyes into the neurons was performed well and whether quantum yields of the dyes were not changed in the intracellular milieu. For that, we calculated ratios of dye fluorescence in the soma (the whole somatic area) and in the pipette (the part of pipette interior in the focal plane 50 μm above the cell soma). It turned out that these ratios were not significantly different (1.45±0.03 vs 1.47±0.03 (mean ± S.E.M.), for cell somas and pipettes, respectively; P = 0.71 pared t-test, n = 8). This demonstrated a good quality of dye perfusion and an absence of shift in quantum yield of the dyes upon washing into the neurons.

Second, a *ratio factor*, reflecting the properties of the equipment and the dyes used was calculated for Oregon Green 488 and Alexa Fluor 594 as demonstrated in Fig 1, which gave the value of 1.32. Dividing the ratio of dye fluorescence in the neuronal soma by the *ratio factor* (1.45/1.32) yields 1.10±0.03, which is, according to Eq 2, the estimate of ratio of Oregon Green 488 to Alexa Fluor 594 concentrations in neuronal soma. This value is reasonably close to 1, a genuine ratio of dye concentrations in the soma.

Thus, the estimate of an error associated with the method obtained in this set of experiments is 10% versus estimated 30% error in the experiments with CTV tandem.

It should be noted, that the accuracy estimated in Oregon/Alexa experiments could be further improved. As some of the Alexa Fluor 594 fluorescence was detected in Oregon Green 488 channel, the correction for this cross-talk has been made, which resulted in 1.04±0.03 estimated ratio of the dye concentrations.

At the same time, we do not consider this improvement feasible to be implemented in practice, since, due to the small masses and volumes involved in dyes preparation, it is technically difficult to obtain concentrations with more than ~10% accuracy (calculations are not shown).

### Measurements of intracellular concentration of fluorescent proteins in diffusionally compact cells

We further employed the approach suggested above in order to measure the concentration of ECFP (enhanced cyan fluorescent protein) in PC12 cells transiently transfected to express this protein. As the reference fluorescence label, we chose Alexa Fluor 594 since it has a negligible spectral overlap with ECFP and therefore fluorescence of the labels can be separated without the need in linear unmixing. In order to load Alexa Fluor 594 (200 μM) into the cells we employed standard patch clamp technique in a whole-cell configuration.

**Fig 3:**
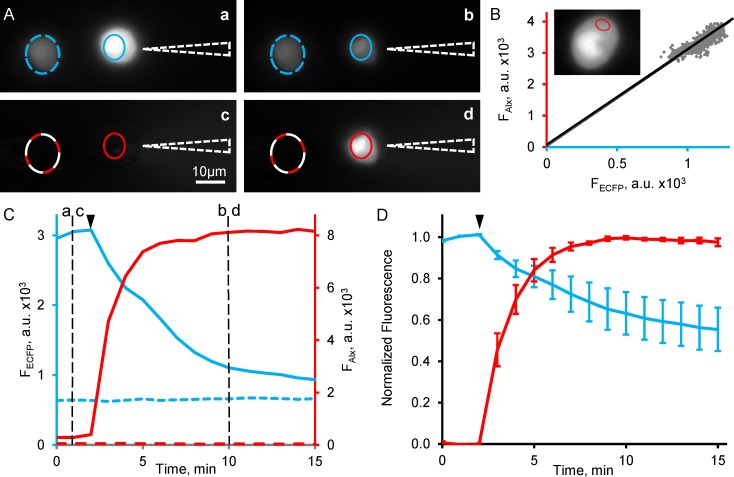
Estimation of ECFP concentration in PC12 cells. ECFP expressing PC12 cells were filled with Alexa Fluor 594 via patch pipette. (A) Images of two PC12 cells obtained in ECFP (a, b) and Alexa Fluor 594 (c,d) channels right before the cell membrane rupture (a, c) and in 10 min after the rupture (b, d). Images were taken at the moments indicated by black dashed lines in (C). A position of patch pipette are depicted in (A) by white dotted triangles. Scale bar is 20 μm. (B) A correlation plot of ECFP and Alexa Fluor 594 fluorescence intensities for a cytoplasmic part of cell. Each dot in the correlation plot represents a pixel located in a red oval depicted within the cytoplasmic part of cell, of which an image is shown in the insert (ECFP channel). ECFP and Alexa Fluor 594 images used for building the correlation plot were obtained by averaging all frames in ECFP channel taken before the cell rupture and all frames in Alexa Fluor 594 channel between 10 and 15 min. A linear regression without offsets for this plot indicates to reasonable correlation between the fluorescent values (R^2^ = 0.67). (C) Time courses of cell loading with Alexa Fluor 594 (red traces) and washing out of ECFP (blue traces). Fluorescence intensities for both labels were calculated in ROIs shown in (A) as an average intensity of all pixels within each ROI. Solid and dashed lines in ROIs (A) and time courses (C) correspond to the patched and intact cell, correspondingly. Cell rupture is indicated by black arrowheads in (C) and (D). Alexa Fluor 594 fluorescence (*F*_*Alx*_) reached a plateau in about 5 min after the membrane rupture indicating an equilibrium state at which the concentration of Alexa Fluor 594 inside the cell and in the patch pipette are expected to be equal. Residual fluorescence in ECFP channel indicates that some ECFP molecules (about 30%) are retained in intracellular compartments. Images were taken at the rate of 1 min per frame for each channel in order to minimize photo-bleaching. ECFP photobleaching in a control cell at this frame rate was negligible (dashed blue trace in Fig 3B); thus, it could be also neglected in the patched cells. (D) Time courses from five cells were normalized and averaged, representing a perfusion dynamics of Alexa Fluor 594 (red traces) and washing out of ECFP (blue traces), analogous to those in (C).

First, we chose a field of view with two PC12 cells transfected with ECFP (Fig 3A). The cell on the left was intact to monitor photobleaching throughout the experiment. The cell on the right was initially patched with the pipette containing known concentration of Alexa Fluor 594 (a reference label) in a cell-attached mode (without rupturing the plasma membrane) and wide field fluorescence recordings in ECFP and Alexa Fluor 594 emission channels were immediately started with a slow rate of one frame per min (Fig 3C). After taking 3 frames the membrane of patched cell was ruptured allowing Alexa Fluor 594 to perfuse into and ECFP to wash out from the cell cytosol. In 5–10 min after the rupture both Alexa Fluor 594 and ECFP fluorescence intensities reached steady-state levels (Fig 3C) indicating that Alexa Fluor 594 concentrations in the pipette and cytosol became equilibrated and ECFP was washed out from the cytosol. Pooled results of such experiments obtained in other cells taken from two different cultures are shown in Fig 3D.

First 3 frames in ECFP channel taken before the membrane rupture, and 5 frames in ECFP and Alexa Fluor 594 channels, taken at steady-state, were averaged and used for the further analysis (ECFP-control, ECFP-plateau and Alexa-plateau, correspondingly). The ECFP-control and Alexa-plateau images were used to determine ratios of these dye fluorescence intensities and to calculate ECFP concentration according to Eq 2. An estimation of the fluorescence intensity ratio can be obtained from a linear regression of pixel-by-pixel correlation plot for a given ROI in the cellular cytoplasm as it is shown in Fig 3B (a red shape in the insert;). It is clearly seen that the pixel-by-pixel correlation is well fitted by linear regression without offsets (R^2^ = 0.67, slope $\frac{F_{ECFP}}{F_{Alexa}}=0.33$). Alternatively, the ratio could be also estimated by dividing mean values of ECFP and Alexa Fluor 594 fluorescence from the regions of interest in ECFP-control and Alexa-plateau images, respectively ($\frac{F_{ECFP}}{F_{Alexa}}=0.33$). Thus, both types of simple calculations, the linear regression of correlation plot and the division of fluorescent intensities in the ROIs, can be used to estimate a ratio of label fluorescence intensities. Next, we calculated the *ratio factor A*, specific for ECFP and Alexa Fluor 594 pair of labels in the way demonstrated for Cerulean and Venus labels above (Fig 1). Its value was calculated to be 18.6. An effective cytoplasmic ECFP concentration estimated for the representative cell (Fig 3B) using the values of fluorescence intensity ratio and the *ratio factor* was

$${[L}_{ECFP}]=\frac{1}{A}\frac{F_{ECFP}}{F_{Alexa}}[L_{Alexa}]=\frac{1}{18.6}*0.33*200\mu M=3.51\mu M$$

The concentration in the somatic cytoplasm of all tested PC12 cells was estimated as 4.01±1.68 μM (median with interquartile range, n = 5).

The proposed method implicitly assumes that the fluorophores, whose fluorescence are analyzed, occupy the same volume. In all tested PC12 cells (Fig 3C and 3D), ECFP could not be washed out completely from the cell indicating that a significant part of ECFP was retained within intracellular compartments (e.g. the endoplasmic reticulum or Golgi apparatus) that were inaccessible to a normal diffusion process. On average, upon washout, ECFP fluorescence intensity was reduced by 44±7% (n = 5) compared to its initial level. On the other hand, Alexa Fluor 594, a strongly charged molecule, cannot enter intracellular compartments and acquires a completely cytosolic distribution. Therefore, in order to obtain an estimation of cytosolic (rather than effective cytoplasmic) ECFP concentration, only the cytosolic portion of ECFP has to be taken into account. This is done by subtracting the ECFP-plateau image (taken when only compartmentalized ECFP is supposed to remain within the cell) from the initial ECFP-control image taken before cell rupture (representing both cytosolic and compartmentalized localization of ECFP). This differential image, therefore, represents the cytosolic ECFP localization while Alexa-plateau image represents the cytosolic Alexa Fluor 594 localization in the same volume. For the cell demonstrated in Fig 3B, a ratio obtained for the same region of interest (the red shape in the insert) by dividing the differential ECFP image and Alexa Fluor 594 image ($\frac{F_{ECFP}}{F_{Alexa}}=0.20$) resulted in the cytosolic ECFP concentration of 2.14 μM in this particular cell (compared to the effective cytoplasmic concentration of 3.51 μM estimated above). We have also estimated (see Methods) for all tested cells that mobile (most probably cytosolic) and immobile (mainly expressed in the intracellular compartments) fractions of ECFP were 44% and 56% (Fig 3C), respectively, yielding 1.76±0.87 μM (median with interquartile range, n = 5) as the estimation of concentration for the cytosolic ECFP fraction.

Our results demonstrate that a complete loading of compact cells with a reference label and washing out cytosolic fraction of the target label via a patch pipette can be accomplished in about 5 min. Thus, recording cell images in the reference and target label channels before and 5 min after cell membrane rupture allows one to estimate the effective and cytosolic concentrations of the target label and to quantitatively analyze its distribution between the cytosol and intracellular compartments.

### Measurements of intracellular concentration of exogenous hippocalcin in neurons

We further employed the suggested approach in order to estimate the concentration of neuronal Ca^2+^ sensor protein, hippocalcin, tagged by EYFP, HPCA-EYFP, in a dendritic tree of hippocampal neurons transiently transfected to exogenously express this protein. Hippocalcin participates in Ca^2+^-dependent signaling in dendrites [6,23] and it is important to verify that intrinsic hippocalcin signaling in the hippocampal neurons is not substantially disrupted by the exogenously expressed HPCA-EYFP. We assumed that the influence of exogenous HPCA-EYFP on the function of the endogenous hippocalcin would be negligible if its concentration does not exceed a half of endogenous hippocalcin concentration known to be around 30 μM [24]. Thus, our goal was to estimate dendritic HPCA-EYFP concentration in order to have a possibility in the future experiments to select hippocampal neurons expressing an appropriate level of HPCA-EYFP. As the reference fluorescence label, we chose Alexa Fluor 594 as in the previous series of experiments.

**Fig 4:**
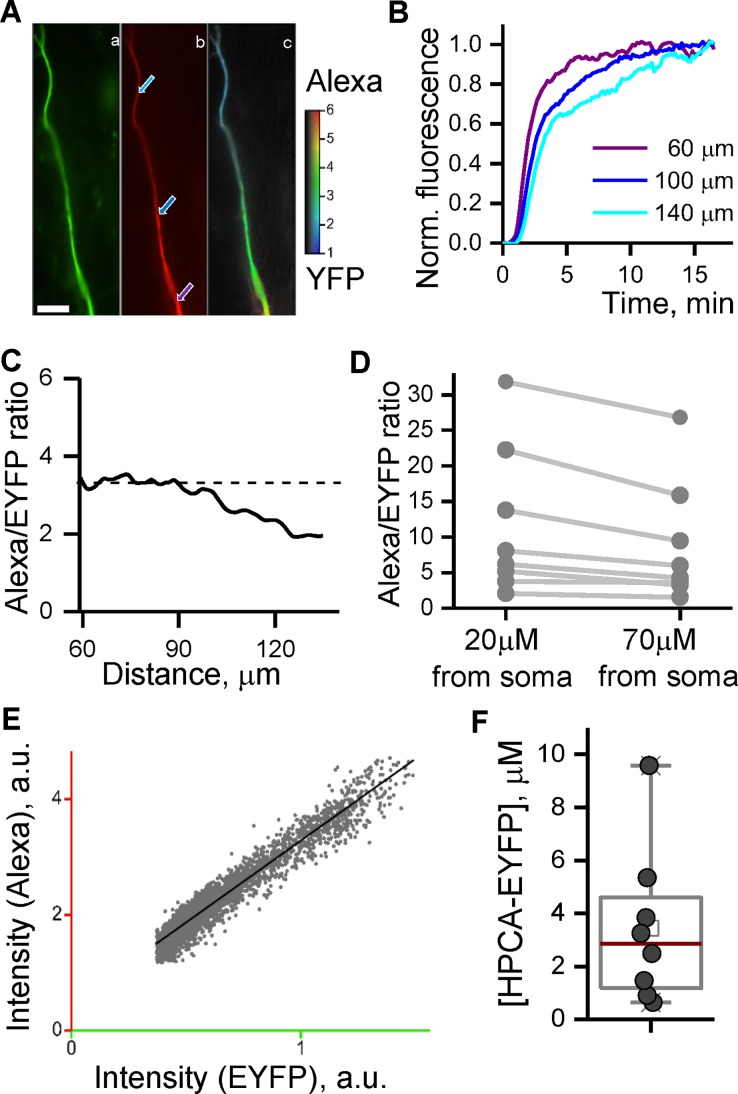
Estimation of HPCA-EYFP concentration in a dendritic tree of cultured hippocampal neurons. Hippocampal neurons (DIV 10–20) expressing HPCA-EYFP were patched with pipettes filled with an intracellular solution containing Alexa Fluor 594 dye (20–50 μM). (Aa) HPCA-YFP image of dendrite taken at the beginning of experiment, before the membrane rupture. (Ab). Alexa Fluor 594 image of the same area taken in 15 min after cell rupture. Analogous images were taken in other tested neurons and used for obtaining graphs shown in (D) and (F). Arrows denote regions of interest, ROIs, where the fluorescence intensity was measured; the corresponding time courses shown in (B). (Ac). A color-coded ratio of Alexa Fluor 594 to HPCA-YFP fluorescence intensities. Scale bar is 10μm. (B) Intensity of Alexa Fluor 594 fluorescence was measured over time at ROIs indicated by the arrows in (Ab). Intensity profiles are normalized by their maximal values. Colors of the traces correspond to the colors of the arrows in (Ab). (C) A ratio profile of Alexa Fluor 594 to HPCA-YFP fluorescence taken along the dendrite. The plot demonstrates a plateau region up to 100μm from soma where Alexa Fluor 594 wash-in was competed in 15 min after the membrane rupture thus far indicating a range of distances from soma where HPCA-EYFP concentration can be accurately estimated. (D) Ratios of Alexa Fluor 594-to-HPCA-EYFP fluorescence in the proximal and distal parts of the apical dendrites in 15 min after the membrane rupture for 8 tested neurons. The graph demonstrates substantial wash-in of Alexa Fluor 594 in the distal parts of dendrites. A ratio of Alexa/EYFP fluorescence in a distal part normalized to the same ratio in a proximal part was 0.75±0.04. (E) A correlation plot of HPCA-EYFP and Alexa Fluor 594 fluorescence intensities generated for all pixels in the dendrite corresponding to the plateau region in (C) (green region in (Ac)). A linear regression of this plot indicates to a significant linear correlation between HPCA-YFP to Alexa Fluor 594 fluorescence (R^2^ = 0.87). (F) Estimated dendritic concentrations of HPCA-EYFP in the cytosol pulled from eight tested neurons.

First, HPCA-EYFP fluorescence was recorded in a long apical dendrite of the neuron (Fig 4Aa). Secondly, we loaded the neuron with Alexa Fluor 594 via a patch pipette in order to introduce a reference fluorescent label into the apical dendrite (Fig 4Ab). Practice evidences that perfusion of branched cells may take considerable time. To be used as a reference, the concentration of fluorescent label has to be stabilized after a cell rupture in the area where the target label concentration is to be estimated. We tested the characteristic time needed for Alexa Fluor 594 concentration to settle, in proximal and distal parts of apical dendrite of the hippocampal neuron in patch clamp conditions. In representative experiments (access resistance of 15 MOhm) spatial distribution of Alexa Fluor 594 fluorescence intensity in the long apical dendrite reached a steady-state level in about 15 min after the membrane rupture at distances up to 100 μm from a soma (Fig 4B). A ratio image of Alexa Fluor 594 to HPCA-EYFP fluorescence (Fig 4Ac) and a ratio profile along the dendrite (Fig 4C) demonstrate that the concentration of Alexa Fluor 594 in the dendritic segment 60 to 100 μm away from a cell body was equilibrated with the Alexa concentration in the patch pipette.

However, at the distal part of the apical dendrite (Fig 4Ac, 4B and 4C) wash-in of Alexa was not complete. Polled results demonstrate (Fig 4D) that a ratio of Alexa-to-HPCA-EYFP fluorescence ratios in the proximal and distal parts of the apical dendrites in 15 min after the membrane rupture was 0.75±0.04 (n = 8 neurons) being reasonably close to 1 indicating the completion of wash-in of Alexa Fluor 594. Moreover, this ratio was equal to 0.83±0.05 (n = 4) for recordings, in which an access resistance was less than 18MOhm. This indicates that a concomitant error in calculation of fluorescent label ratio at a distance of 70μm from neuronal soma arising from an incomplete wash-in of Alexa Fluor 594 in a short-term (less than 15min) experiments would not exceed 25% and could be further decreased with decreasing of access resistance and increasing a duration of cell perfusion. Thus, a concentration of hippocalcin in distal parts of dendrites can still be estimated with a reasonable accuracy in fast experiments. Conventional patch clamp experiments, usually, last longer than 15 min creating natural conditions for a complete wash-in of reference label and subsequent improvement in accuracy of the method in distal dendrites and axons.

As it has been demonstrated in previous section, there are two approaches to calculate a ratio of target to reference fluorescence intensities. A linear regression of pixel-by-pixel correlation plot for two imaging channels can be calculated for the part of a dendrite, in which Alexa Fluor 594 is diffusionally equilibrated (the green part in Fig 4Ac; Fig 4E, slope $\frac{F_{HPCA-YFP}}{F_{Alexa}}=0.35$, R^2^ = 0.87). Alternatively, a good linear correlation between the fluorescent label intensities prompts the estimation of the ratio by relating mean values of fluorescent intensities in ROIs placed over the dendrite in HPCA-EYFP and Alexa Fluor 594 images ($\frac{F_{HPCA-YFP}}{F_{Alexa}}=0.28$), thus far greatly simplifying the task. The latter approach was used in the following calculations.

Next, we calculated a *ratio factor A*, specific for Alexa Fluor 594 and EYFP labels. We did this in a manner illustrated in previous sections using optical spectra, extinction coefficient, and quantum yield of EYFP instead of ECFP used in the previous section. The *ratio factor* for these labels was 1.02.

Finally, HPCA-EYFP concentration in the dendrite of a representative neuron was estimated as:

$${[L}_{HPCA-EYFP}]=\frac{1}{A}\frac{F_{HPCA-YFP}}{F_{Alexa}}[L_{Alexa}]=\frac{1}{1.02}*0.28*20uM=5.5\;\mu M$$

The concentration in the dendrites of tested neurons was estimated as 3.4±1.1 μM (median with interquartile range, n = 8, Fig 4F).

## Discussion

We have proposed a simple, yet universal technique for measuring intracellular concentration of fluorescent molecules. The method is convenient for a fast estimation of cytosolic fluorophore concentrations at a single cell level. It is mainly applicable to fluorophores preloaded (e.g. cell-permeant dyes) into single cells or fluorescent proteins exogenously expressed in the cells. The method also enables independent measurements of cytosolic fluorophore concentration from different regions within a cell (e.g. soma and cell processes in case of neurons) and estimating mobile and immobile fractions of the fluorophore under study.

### Comparison of the new and previously developed approaches

Several elegant approaches have been previously proposed in order to adequately quantify the expression levels of fluorescent proteins, FPs. Van der Wal et al. compared fluorescence intensity of FP exogenously expressed in cell bodies and purified, bacterially expressed FP in a solution [25]. Alternatively, fluorescence measurements of exogenously expressed FP were calibrated with transparent beads and gels that have known densities of FP [26]. Authors considered that equal levels of fluorescent intensities of exogenous and purified FPs represented their equal concentrations. However, the level of fluorescence recorded from a sample depends upon both fluorophore concentration and the effective volume from which a fluorescent signal is captured: given the concentrations are equal, the larger volume will produce the larger amount of photons. If a wide-field imaging system is used for fluorescence recording, the effective fluorescent volumes may differ between the soma, which fluorescence is contained within the cell outlines, and the bulk of the beads suspension, for which a significant amount of out-of-focus fluorescence is captured along with the relevant signal. In this view, rectangular cross-section glass capillaries of known internal dimensions were suggested as a further improvement in order to measure reference fluorophore volumes and to partially overcome this problem [4,27]. Authors filled the capillaries with a recombinant FP of certain concentration and imaged them to obtain a concentration calibration curve for the protein fluorescence. The calibration curve was used to calculate the total FP concentration within the cell soma based upon fluorescence recorded from individual cells and their soma sizes.

Another approach has been developed which allows comparison of the calibration signal and test signal in the same sample and under identical imaging conditions. M. Dundr et al.[5] relied on the use of rotavirus-like particles introduced to a sample and containing a known number of GFP molecules as an internal calibration standard.

These approaches, however, require producing recombinant protein and preparing fluorescent beads as additional expensive and prolonged steps in experimental settings. More importantly, fluorescent protein properties depend on pH, presence of metal ions and other parameters of the protein microenvironment. Consequently, the fluorescence properties of recombinant protein in a calibration solution may differ from ones of the intracellular target fluorescent protein, which precludes a usage of recombinant protein as a reliable calibration tool.

The other caveat to using recombinant proteins is their post-translational modification in a chosen expression system, since protein tertiary structure directly determines biophysical properties of the protein. E.coli, the most typical hosts, are prokaryotes. Possessing distinct chaperone machinery[28] they may fold protein differently [29]. Disulfide bonds, another factor determining tertiary structure, are also acquired by distinct mechanisms (see Hatahet et al. for review [30]). Furthermore, glycosylation or other covalent modifications inherent to eukaryotes may not be present in the expression system. These differences in molecular structure may result in a substantial difference in fluorescence properties of exogenously expressed and recombinant proteins leading to an additional error in estimation of FP concentration.

Finally, and most importantly, all previously developed approaches did not consider that volumes, from which a reference and target fluorescence was collected, were intrinsically (structurally) different. For example, a bead or a part of capillary could have the same volume as a cell soma; however, the volume of cytosol, in which the target fluorophore is distributed, is smaller than the total cell volume that could result in substantial (many fold) errors in estimation of target fluorophore concentration.

In spite of clear leads to possible errors, no procedures have been suggested for error estimations in the previously developed approaches. The approach presented in this work is based on usage of whole cell patch clamp technique [22], a commonly employed electrophysiological method developed to precisely control a content of intracellular solution and to record transmembrane currents. Particularly, this allowed to control pH, ionic strength and composition of intracellular environment in our experiments at values close to those, at which spectral properties of the fluorescent labels had been obtained. Furthermore, using the intracellular perfusion we were able to introduce a reference label of known concentration to exact location of the target label in the cytosol that has substantially improved the reliability and accuracy of the measurements. This has minimized errors related to improper estimation of volumes occupied by reference and target labels, immanent to other methods, and allowed to estimate fluorophore concentrations in small subcellular structures (Fig 4).

Sequential recordings of the reference and target fluorescence minimized an interaction between the fluorophores rendering FRET and other related issues impossible between the reference and target labels (Figs 3 and 4) and allowing to utilize fluorophores with partially overlapping spectra.

Without attempting to address every possible source of error we have designed and performed, for the first time, a validation procedure to estimate an “integral” error of the method. Experiments with a Cerulean and Venus tandem described in Fig 2 explicitly estimates the error originating from a combination of all possible factors. The compound error has been estimated to be about 30% (Fig 2D) for the case of two fluorescent proteins present in a cell. However, the tandem measurements include many theoretical assumptions, which may contribute to the observed error value. Performing experiments, in which two organic dyes were loaded to cells in known concentrations, we demonstrated that the error in an estimation of fluorescent label concentration can be less than 10%. Towards additional improvement of accuracy, spectra of fluorescent proteins within cells can be measured in the same experiment and used instead of the data obtained from suppliers. In this way any environment-dependent modifications of fluorescent label spectra can be corrected.

### Technical implementation of proposed approach

The main assumption of the method is that both the reference fluorophore and the fluorophore whose concentration is being analyzed (target fluorophore) are spatially co-distributed, i.e. their concentrations ratio is constant throughout the volume of interest. In our experimental approach this requirement is fulfilled for all cellular moieties diffusionally coupled with the cytoplasm (and hence, with a patch pipette when the plasma membrane is ruptured). There are cases, however, when a significant part of the target protein is confined in the endoplasmic reticulum or Golgi apparatus, wherein undergoing sorting and posttranslational modifications until being released to the cytosol. This compartmentalized immobilized part of the protein will contribute to a recorded fluorescent signal although it is cytoplasmic rather than cytosolic. Since the strongly charged reference fluorophore cannot reach intracellular compartments, the cytosolic concentration of target FP based on target-to-reference fluorescence ratio (Eq 2) will be overestimated.

Fortunately, the immobile part of the target fluorophore can be easily quantified for further correction by subtracting the images acquired before and after target fluorophore washout (see Methods section and Fig 3C and 3D).

The other point that should be considered is a sample thickness compared to a focal depth of the objective used for imaging. Fluorescence from thin (1–3μm) objects, like dendrites and axons, are almost fully captured using wide field microscopy having the same focal depth of 1–2 μm. In this case, fluorescence emitted by all reference and target dye molecules in the sample is collected in the respective imaging channels and the ratio of dye fluorescence faithfully reflects the ratio of their amounts. When both fluorophores are cytosolically distributed the fluorescence ratio faithfully represents the ratio of fluorophore concentrations. In larger objects, such as neuronal soma, only 1–2 μm thick optical slice would contribute to a recorded fluorescent signals in both reference and target fluorophore channels. In this case an estimation of target fluorophore concentration can be obtained for the particular optical slice within the cell under study. The use of small aperture objectives having higher focal depth could be appropriate in this case in order to estimate an averaged target fluorophore concentration in the somatic cytosol. On the other hand, using confocal microscopy would allow to estimate not only a cytosolic FP concentration but a spatial 3D profile of target fluorophore distribution between the cytosol and intracellular compartments.

The need for spectral data for every optical element of the imaging system may seem burdensome, however, in most cases, filters, mirrors and other optical parts come with a certificate of analysis, which describes spectral properties of a part. Moreover, once collected it can be used through the whole life cycle of the imaging system. Also, a typical filter cube with a dichroic mirror (so called, 3-cube) having 3 excitation and emission bands can be used for a variety of fluorescent labels; therefore, a single calculation of the equipment optical function for excitation and emission light paths (Fig 1A and 1C) can be used for many different pairs of fluorescent labels.

We should stress that the accuracy of the *ratio factor* calculations depends on the accuracy of the manufacturer-supplied data describing spectral properties of the optical parts and the estimated quantum yield of fluorophores. In some cases, spectral properties of optical parts are provided as averaged for a number of identical parts (e.g. for microscope objectives). Thus, some inaccuracy (of about 5%) in calculations of the *ratio factor* is inevitable, so is the consequent error in the calculated target label concentration. It is, of course, advisable to keep all optical elements clean and fixed tightly in their holders and frames to minimize possible deviations in their spectral properties.

However, even these small possible inaccuracies can be minimized if the experimental arrangement and the fluorescent labels are selected appropriately. In an ideal case, if the target and reference labels can be excited and recorded at the same wavelengths, possible inaccuracy in the equipment optical functions cancel out when the *ratio factor* is calculated (Eq 2). At the same time, the larger the difference in wavelengths (used for excitation and emission) between the target and reference labels, the more the *ratio factor* is impacted by possible inaccuracy in the equipment optical functions (calculated based on the provided spectral data). Therefore, it is advisable to choose target and reference labels with spectra as close as possible (for both absorption and emission). Obviously, this makes practical sense only in case when the cell is diffusively compact and the target protein can be easily washed out. In such a case, when the fluorescence of target label has been recorded, the cell is patched and perfused with the reference label. Simultaneously with perfusion, the target label is washed out from the cell. Upon equilibration, the fluorescence of reference label can be recorded.

On the other hand, for the case of branched cells with long processes, it is not always possible to completely washout the target label even if it is not anchored within a cell. In such case, excitation or emission of the target and reference labels should be chosen to be fully separable via available spectral filters. If spectral overlap between fluorescent labels is unavoidable, various mathematical unmixing techniques [10,11,20] can be used in order to separate fluorescent signals of target and reference labels.

### Estimation of hippocalcin concentration in hippocampal neurons

Using the approach suggested in this work we could promptly estimate the dendritic cytosolic concentration of neuronal Ca^2+^ sensor protein, hippocalcin, tagged by EYFP, HPCA-EYFP, which was exogenously expressed in cultured hippocampal neurons. We have demonstrated that a protein expression level substantially varied from cell to cell but did not exceed 10 μM (Fig 4). Thus, the concentration of exogenous hippocalcin in the hippocampal neurons under study is at least 3 times lower compared to the concentration of endogenous one estimated to be about 30 μM [24]. Thus, the exogenous protein may be used as a tool to visualize Ca^2+^-dependent hippocalcin translocation and target interaction [6,23] without substantial perturbation of endogenous hippocalcin signaling

It is important to note that a certain amount of HPCA-EYFP can be located in the dendritic plasma membrane rather than in the cytosol [6] leading to some inaccuracy in estimating of the cytosolic concentration. At the same time, HPCA-EYFP is mainly distributed in the cytosol at low cytosolic Ca^2+^ concentration [6,23] thus far making the estimate of effective HPCA-EYFP concentration close to the cytosolic one. An objective focal depth of wide-field microscopes used in this study (1–2 μm) is close to dendritic diameters (1–3 μm). Thus, the objective collected fluorescence from the whole dendritic depth and even in the case of partial membranous localization of HPCA-EYFP the suggested method would give the correct estimation of total protein concentration in the dendritic tree.

## References

[1] Chalfie M, Tu Y, Euskirchen G, Ward WW, Prasher DC. Green fluorescent protein as a marker for gene expression. Science. 1994;263: 802–805. 8303295 10.1126/science.8303295

[2] Zlokarnik G, Negulescu PA, Knapp TE, Mere L, Burres N, Feng L, et al Quantitation of transcription and clonal selection of single living cells with beta-lactamase as reporter. Science. 1998;279: 84–88. 9417030 10.1126/science.279.5347.84

[3] Phair RD, Misteli T. Kinetic modelling approaches to in vivo imaging. Nat Rev Mol Cell Biol. 2001;2: 898–907. doi: 10.1038/35103000 11733769 10.1038/35103000

[4] Furtado A, Henry R. Measurement of green fluorescent protein concentration in single cells by image analysis. Anal Biochem. 2002;310: 84–92. 12413477 10.1016/s0003-2697(02)00281-6

[5] Dundr M, McNally JG, Cohen J, Misteli T. Quantitation of GFP-fusion proteins in single living cells. J Struct Biol. 2002;140: 92–99. 12490157 10.1016/s1047-8477(02)00521-x

[6] Dovgan A V, Cherkas VP, Stepanyuk AR, Fitzgerald DJ, Haynes LP, Tepikin A V, et al Decoding glutamate receptor activation by the Ca2+ sensor protein hippocalcin in rat hippocampal neurons. Eur J Neurosci. 2010;32: 347–358. doi: 10.1111/j.1460-9568.2010.07303.x 20704590 10.1111/j.1460-9568.2010.07303.x PMC3069492

[7] Thaler C, Koushik S V, Blank PS, Vogel SS. Quantitative multiphoton spectral imaging and its use for measuring resonance energy transfer. Biophys J. 2005;89: 2736–49. doi: 10.1529/biophysj.105.061853 16040744 10.1529/biophysj.105.061853 PMC1366774

[8] O’Callaghan DW, Ivings L, Weiss JL, Ashby MC, Tepikin A V., Burgoyne RD. Differential Use of Myristoyl Groups on Neuronal Calcium Sensor Proteins as a Determinant of Spatio-temporal Aspects of Ca 2+ Signal Transduction. J Biol Chem. 2002;277: 14227–14237. doi: 10.1074/jbc.M111750200 11836243 10.1074/jbc.M111750200

[9] Sondek J, Bohm A, Lambright DG, Hamm HE, Sigler PB. Crystal structure of a G-protein beta gamma dimer at 2.1A resolution. Nature. 1996;379: 369–74. doi: 10.1038/379369a0 8552196 10.1038/379369a0

[10] Wlodarczyk J, Woehler A, Kobe F, Ponimaskin E, Zeug A, Neher E. Analysis of FRET Signals in the Presence of Free Donors and Acceptors. Biophys J. 2008;94: 986–1000. doi: 10.1529/biophysj.107.111773 17921223 10.1529/biophysj.107.111773 PMC2186232

[11] Leavesley SJ, L Britain A, Cichon LK, Nikolaev VO, Rich TC. Assessing FRET using spectral techniques. Cytom Part A J Int Soc Anal Cytol. 2013; doi: 10.1002/cyto.a.22340 23929684 10.1002/cyto.a.22340 PMC4374658

[12] Koushik S V, Vogel SS. Energy migration alters the fluorescence lifetime of Cerulean: implications for fluorescence lifetime imaging Forster resonance energy transfer measurements. J Biomed Opt. 2008;13: 031204 doi: 10.1117/1.2940367 18601528 10.1117/1.2940367 PMC2556851

[13] Nikon Instrumrnts. Introduction to Fluorescent Proteins. In: Nikon MicroscopyU [Internet]. 2016 Available: https://www.microscopyu.com/techniques/fluorescence/introduction-to-fluorescent-proteins

[14] Nagai T, Ibata K, Park ES, Kubota M, Mikoshiba K, Miyawaki A. A variant of yellow fluorescent protein with fast and efficient maturation for cell-biological applications. Nat Biotechnol. 2002;20: 87–90. doi: 10.1038/nbt0102-87 11753368 10.1038/nbt0102-87

[15] Alexa Fluor Dyes Spanning the Visible and Infrared Spectrum—Section 1.3. In: Molecular Probes Handbook. 2016.

[16] Lakowicz Joseph R. Principles of Fluorescence Spectroscopy. 3rd ed. Springer US; 2006 doi: 10.1007/978-0-387-46312-4

[17] Gell C, Brockwell D, Smith A. Handbook of Single Molecule Fluorescence Spectroscopy. Oxford University Press; 2013.

[18] Chroma Technology. Chroma Spectra Viewer. In: Chroma Technology [Internet]. 2016 Available: https://www.chroma.com/spectra-viewer

[19] ThermoFisher Scientific. Fluorescence SpectraViewer. In: ThermoFisher Scientific [Internet]. 2016 [cited 20 Dec 2016]. Available: https://www.thermofisher.com/ua/en/home/life-science/cell-analysis/labeling-chemistry/fluorescence-spectraviewer.html

[20] Dinant C, van Royen ME, Vermeulen W, Houtsmuller AB. Fluorescence resonance energy transfer of GFP and YFP by spectral imaging and quantitative acceptor photobleaching. J Microsc. 2008;231: 97–104. doi: 10.1111/j.1365-2818.2008.02020.x 18638193 10.1111/j.1365-2818.2008.02020.x

[21] Hamill OP, Marty A, Neher E, Sakmann B, Sigworth FJ. Improved patch-clamp techniques for high-resolution current recording from cells and cell-free membrane patches. Pflugers Arch. 1981;391: 85–100. Available: http://www.ncbi.nlm.nih.gov/pubmed/6270629 6270629 10.1007/BF00656997

[22] Molleman A. Patch Clamping [Internet]. Chichester, UK: John Wiley & Sons, Ltd; 2002 doi: 10.1002/0470856521

[23] Markova O, Fitzgerald D, Stepanyuk A, Dovgan A, Cherkas V, Tepikin A, et al Hippocalcin signaling via site-specific translocation in hippocampal neurons. Neurosci Lett. 2008;442: 152–7. doi: 10.1016/j.neulet.2008.06.089 18634855 10.1016/j.neulet.2008.06.089 PMC2572729

[24] Furuta Y, Kobayashi M, Masaki T, Takamatsu K. Age-related changes in expression of hippocalcin and NVP2 in rat brain. Neurochem Res. 1999;24: 651–8. Available: http://www.ncbi.nlm.nih.gov/pubmed/10344594 10344594 10.1023/a:1021000425070

[25] van der Wal J, Habets R, Várnai P, Balla T, Jalink K. Monitoring agonist-induced phospholipase C activation in live cells by fluorescence resonance energy transfer. J Biol Chem. 2001;276: 15337–15344. doi: 10.1074/jbc.M007194200 11152673 10.1074/jbc.M007194200

[26] Chiu C-S, Jensen K, Sokolova I, Wang D, Li M, Deshpande P, et al Number, density, and surface/cytoplasmic distribution of GABA transporters at presynaptic structures of knock-in mice carrying GABA transporter subtype 1-green fluorescent protein fusions. J Neurosci Off J Soc Neurosci. 2002;22: 10251–10266. 10.1523/JNEUROSCI.22-23-10251.2002 PMC6758747 12451126

[27] Hack NJ, Billups B, Guthrie PB, Rogers JH, Muir EM, Parks TN, et al Green fluorescent protein as a quantitative tool. J Neurosci Methods. 2000;95: 177–84. Available: http://www.ncbi.nlm.nih.gov/pubmed/10752489 10752489 10.1016/s0165-0270(99)00178-8

[28] Carrascosa JL, Llorca O, Valpuesta JM. Structural comparison of prokaryotic and eukaryotic chaperonins. Micron (Oxford, Engl 1993). 2001;32: 43–50. 10.1016/s0968-4328(00)00027-5 10900379

[29] Gething MJ. Protein folding. The difference with prokaryotes. Nature. 1997;388: 329, 331. doi: 10.1038/40979 9237748 10.1038/40979

[30] Hatahet F, Boyd D, Beckwith J. Disulfide bond formation in prokaryotes: History, diversity and design. Biochim Biophys Acta. 2014; doi: 10.1016/j.bbapap.2014.02.014 24576574 10.1016/j.bbapap.2014.02.014 PMC4048783

